# Introducing Advanced Paramedics into the rural general practice team in Ireland – general practitioners attitudes

**DOI:** 10.1186/s12875-022-01740-9

**Published:** 2022-05-26

**Authors:** F. Feerick, C. O. Connor, P. Hayes, D. Kelly

**Affiliations:** 1 School of Medicine, University of Limerick, Limerick, Ireland; 2 Health Research Institute, University of Limerick, Limerick, Ireland

**Keywords:** Advanced paramedic, General practice, General practitioners, Primary care

## Abstract

**Introduction:**

As Ireland's population increases and chronic disease becomes more prevalent, demand on limited general practice services will increase. Nursing roles within general practice are now considered to be standard, yet alternative allied health professional roles are under explored within an Irish context. Allied health personnel such as Advanced Paramedics (APs) may have the capability to provide support to general practice.

**Aim:**

To explore General Practitioners’ (GPs) attitudes and opinions of integrating Advanced Paramedics (APs) into rural general practice in Ireland.

**Methods:**

A sequential explanatory mixed methodology was adopted. A questionnaire was designed and distributed to a purposeful sample of GPs attending a rural conference followed by semi-structured interviews. Data was recorded and transcribed verbatim and thematically analysed.

**Results:**

In total *n* = 27 GPs responded to the survey and *n* = 13 GPs were interviewed. The majority of GPs were familiar with APs and were receptive to the concept of closely collaborating with APs within a variety of settings including out-of-hours services, home visits, nursing homes and even roles within the general practice surgery.

**Conclusion:**

General Practitioner and Advanced Paramedic clinical practice dovetail within many facets of primary care and emergency care. GPs recognise that current rural models are unsustainable and realise the potential of integrating APs into the general practice team to help support and sustain the future of rural general practice services in Ireland. These interviews provided an exclusive, detailed insight into the world of general practice in Ireland that has not been previously documented in this way.

**Supplementary Information:**

The online version contains supplementary material available at 10.1186/s12875-022-01740-9.

## Introduction

General practice in Ireland may be unsustainable into the future [1]. The health needs of an increasing population are becoming more complex as people are now living longer with chronic disease and co-morbidity [2]. Ireland's demographic and life expectancy is gradually increasing year on year with 4.9 million people now living in Ireland- an increase of 64,500 since 2017 [3]. Projections estimate a population increase to 6.729 million people, with significant increase within the over 65 s group, which will rise between 1.392 and 1.451 million by 2046 with trends forecast to continue over the coming years [4]. Increased population, life expectancy and ageing will place greater demands on our health services [5]. More services are moving towards general practice and primary care as government policy shifts services away from the traditional acute hospital setting [6]. General Practitioner numbers are not sufficient to meet the current and predicted increase in service demand [7]. Though Ireland has increased its graduate intake to medical schools over the past number of years there is a shortage of physicians within the Irish health system with an over reliance on foreign trained doctors to fill the void [8]. Ireland is facing a shortfall of non-consultant hospital doctors (NCHD), physicians and general practitioners with predictions that general practice will have 1800 less GPs than the European Union (EU) average by 2021 [9]. Healthcare workforce shortage is a global problem especially within the areas of nursing and medicine, especially in rural areas [10, 11]. There is also an estimated 2.3 million world-wide shortage of nurses, midwives, and physicians especially within developing countries and low paid economies [12, 13]. Domestic markets will struggle to train and retain sufficient healthcare workers to meet the growing demands on health services in the future [14]. To help alleviate medical workforce shortage, other jurisdictions have redistributed tasks among allied healthcare workers to reduce the burden on limited doctor availability [15, 16]. Ireland has been slow to embrace these innovative practices, maintaining tradition over necessity. Advanced Nurse Practitioners and Physicians Assistants are forms of task redistribution or task shifting where roles such as advanced diagnostics and medical prescribing that was once solely the domain of the Doctor, are successfully subsumed by alternative healthcare workers [17–19]. Internationally, paramedics have evolved into a variety of healthcare settings, including general practice, acute hospital trusts, forensic health care, minor injury units and urgent care units [20–22], however the potential role of the paramedic has not yet been fully explored within an Irish context. The aim of this study therefore was to explore General Practitioners’ attitudes and opinions of integrating Advanced Paramedics into rural general practice in Ireland.

### Irish health system

Ireland does not have universal registration with a General Practitioner. Almost 45% of the population is registered through the Primary Care Reimbursement Service (PCRS), with the remainder being considered private patients and able to attend any GP [23]. Most patients aged over seventy years, all patients under six years, and those below defined income levels are entitled to be registered with the PCRS and entitled to a free GP visit card or further support depending on their level of income (hospital charges, medications fees can also be covered etc.). Practices are funded via fees from private patients (non-PCRS patients) per appointment, fixed capitation payments per patient registered with the PCRS, and various other schemes (cervical smear program, vaccination programs, chronic disease management programs, etc.) [24]. Reimbursement for Advanced Paramedics in General Practice does not currently exist in Ireland, as they are funded through the National Health Service and the National Ambulance Service (NAS). The NAS is the statutory pre-hospital emergency and intermediate care provider for the Irish State. Advanced Paramedics are state employees and provide an advanced level of clinical care beyond the traditional Paramedic, including advanced life support, intubation, and intravenous cannulation, with extended medications including analgesics, antibiotics, and benzodiazepines. The Advanced Paramedic practice autonomously with support from clinical practice guidelines (CPGs) and with medical oversight availability that is directly funded by the national Health Service Executive (HSE). Though some ambulance services are provided by private and not-for-profit providers, they are not predominantly employed by the state to provide emergency services [25].

## Methods

### Study design

Data was collected over one year within two separate phases. Phase one, (quantitative phase, pre-covid). The quantitative data informed phase 2, the qualitative enquiry by supporting the development of the semi-structured interviews to develop and expand its meaning by establishing the attitudes and opinions of General Practitioners towards introducing Advanced Paramedics into General Practice [26]. This study is grounded in the mixed methodologies underpinned by a pragmatic paradigm as described by Feilzer, 2010, that utilises the strengths of both quantitative and qualitative data collection methods [27]. A Sequential explanatory strategy was utilised for data collection purposes where quantitative data was collected within the first phase and qualitative data collected within the second phase [28]. It was felt that one to one interview with GPs were the best method to assess opinions on the research question. Focus groups were considered but were discounted at this stage-primarily because of public health guidelines advising against mass gatherings. The cost of arranging these meetings in an online and recordable format was also prohibitive as this study was not funded.

### Quantitative data collection

A suitable pre-existing validated survey questionnaire could not be found therefore a questionnaire was designed and validated through a process of pilot study and expert peer review panel [29]. The expert panel consisted of two experienced GPs, a Senior Lecturer in Paramedicine, and a Paramedicine research fellow. The questionnaire was piloted using a convenience sample of fourteen General Practitioners attending a one-day training course in the University of Limerick, Medical School. A paper-based copy of the questionnaire was distributed to attendees and 20 min were then dedicated to feedback and informal discussion to establish their opinion on the questionnaire content and design, particularly on the meaning of questions and wording. Comments were noted and the expert panel met and revised the survey instrument with the feedback. The questionnaire design was closely guided by previous works by Bickman, et al., (2008) [26]. The questionnaire was distributed to a purposeful sample of GPs attending a General Practitioners rural medical conference in the midlands of Ireland.

### Qualitative data collection

Comprehensive qualitative semi-structured interviews were conducted with General Practitioners working in the Republic of Ireland, (ROI). GPs were recruited via the Irish Rural and Dispensing Doctor mailing lists (http://www.ruraldoctors.ie/). The Rural, Island and Dispensing Doctor’s Institute of Ireland is in existence since 1984, with a focus on providing medical care locally to rural, island and isolated populations. Respondents were invited via mailing list to participate in the study. No gratuity was offered. Respondents emailed FF to express their interest in participating and were then given more information about the study. A suitable time and date for the interview was scheduled. Reminder emails were sent to the mailing list until sufficient respondents were recruited and data saturation and repetition was achieved. Written consent was obtained prior to the interview with participants emailing a completed consent form to FF. The interview guide was developed in collaboration with the research team as the interviews evolved, to explore key aspects in greater depth and meaning. Participants responded from both urban and rural practice across the Republic of Ireland with a large geographical representation presented in Table 3. The focus of the interviews was to explore General Practitioners’ attitudes to the concept of introducing Advanced Paramedics into General Practice in the Republic of Ireland (ROI). The interviews were conducted by the first author with no affiliation to any of the participants and were held online via Zoom platform with the ability to record both audio and visual communications. Field notes were taken. Interviews varied in duration ranging from thirty minutes to ninety minutes and were accurately transcribed verbatim.

### Data analysis

Quantitative survey data was described in table format using numbers and percentages. Data from the interviews was analysed using the six-stage thematic analysis as described by Braun and Clarke (2006) [30]. Qualitative transcription software was utilised to support the data sorting phase (Doc-tools Extract Changes Pro). Three transcripts were selected at random by the senior authorship team and reviewed in isolation and generated initial codes as per step two of the thematic analysis process. Once consensus was reached between the reviewers on the identified codes the remainder of the transcripts were coded, and data collated. A thematic mind-map was developed that provided a clear overview of the emerging themes and relationships between various data sets. Transcripts were revisited several times during this process to define and further refine the themes and subthemes for analysis to identify the essence of what each theme is about [30].

## Results

### Questionnaire

Table 1 provides an overview of the twenty-seven GP respondents that completed the questionnaire.

**Table 1:** GP demographic

			**Total**
Gender	Male (*n* = 17)	Female (*n* = 10)	(*n* = 27)
Age	< 50 years (*n* = 2)	50 – 60 years (*n* = 6)	> 60 years (*n* = 19) (*n* = 27)
Practice type	Urban (*n* = 2)	Rural (*n* = 19)	Mixed (*n* = 6) (*n* = 27)
GP trainer	Yes (*n* = 18)	No (*n* = 9)	(*n* = 27)
Number of patients per practice	< 1000 (*n* = 1)	1000–6000 (*n* = 24)	> 6000 (*n* = 2) (*n* = 27)
Familiarity with AP practice	Familiar (*n* = 15)	Unfamiliar (*n* = 9)	Undecided (*n* = 3) (*n* = 27)

Table 2 Presents a range of questions tabulated in table format. The questions 1–10 posed a variety of clinical presentations and management of these conditions by the AP, and the GPs were asked whether they deemed these tasks transferable. There was strong consensus among the twenty-seven respondents (*n* = 27) in all areas from cardiovascular assessment and management to respiratory, and gastrointestinal clinical presentations.

**Table 2:** GP questionnaire about AP skills transferability to general practice

		**Agree**	**Disagree**	**Undecided**
1	AP performing acute cardiovascular examination	78%	11%	11%
2	AP performing acute cardiovascular management	85%	4%	4%
3	AP performing acute respiratory examination	67%	11%	4%
4	AP performing acute respiratory management	81%	4%	15%
5	AP performing acute gastrointestinal examination	59%	15%	26%
6	AP performing acute gastrointestinal management	59%	15%	26%
7	AP performing acute neurological examination	44%	19%	37%
8	AP performing examination & management in surgery	63%	15%	22%
9	AP performing examination & management in patient’s home on their behalf	67%	11%	22%
10	AP performing recognition and verification of death on their behalf	78%	7%	15%

### Qualitative evaluation of semi-structured interviews

Thirteen GPs were interviewed. The geographical spread of the participants provided a broader insight into the challenges facing rural general practices incorporating nine different counties from the north, south, east, and west of the island, Table 3.

**Table 3:** Participant demographic

Identifier	Gender	Age	Practice	Background
GP01	Male	> 55	Rural	Single-handed practice
GP02	Male	> 55	Mixed	Single-handed practice
GP03	Male	> 55	Mixed	Group practice
GP04	Male	> 55	Rural	Single-handed practice
GP05	Female	< 55	Mixed	Group practice
GP06	Female	< 55	Rural	Single-handed practice
GP07	Male	> 55	Rural	Single-handed practice
GP08	Male	< 55	Mixed	Group practice
GP09	Female	< 55	Mixed	Group practice
GP10	Female	< 55	Rural	Group practice
GP11	Female	< 55	Rural	Group practice
GP12	Male	> 55	Rural	Single-handed practice
GP13	Male	> 55	Rural	Group practice

### Common themes

The three major themes identified from our analysis were, current and future healthcare challenges, primary care and paramedicine partnership and from theory to tangibility as outlined in Table 4. Major themes were divided into a series of subthemes described below.

**Table 4:** Summary of qualitative data in major themes and subthemes

Major Theme	Subtheme
1.Current & Future Healthcare Challenges	i. Sustainability ii. Capacity & Workload iii. Rural vs Urban Practice
2.Primary Care & Paramedicine Partnership	Out-of-Hours Services Primary Care Team General Practice Based Advanced Paramedic
3.From Theory to Tangibility	i. Consensus ii. Communication iii. Innovation iv. Employment v. Barriers and Enablers

### Current & future healthcare challenges

This theme relates to the current and future challenges facing rural general practice. GPs acknowledge workload, burnout, and capacity to deliver services to be a major challenge within its current health-system in the Republic of Ireland (ROI).

i. **Sustainability**
  General Practitioners believe that rural general practice in Ireland is unsustainable and feel that policy makers are not listening to their concerns. GPs feel that incentivising young doctors to rural areas will be an ongoing challenge and that single-handed practices are no longer viable. The burden of running a practice and providing out of hours services and home visits is becoming increasingly difficult.
  “It’s not sustainable, we’re not training nearly enough doctors who want to stay in the system…there’s a lot of towns, small towns, villages, without a GP at all, and that’s only getting worse” (GP04)
ii. **Capacity and Workload**
  With the current model there is a growing fear among General Practitioners that elderly people with chronic disease living in isolation will become even more disenfranchised. An aging GP population was a key concern. Who will replace these GPs? Although the literature describes the challenges facing rural General Practice, respondents felt that this is also an issue for smaller urban practices and for that in border counties. A common theme among respondents is that they described a detachment between GPs and policy makers and the HSE where they expressed a sense of frustration. Capacity and workload were a major theme among respondents; this was highlighted several times. A new trend described among GPs was that they are seeing more patients that may not necessarily need to be seen at all—“*the worried well*”. This is also impacting on their ability to treat the patients that do require their care. General Practitioners describe the burden of unscheduled emergencies and the impact that these can have on the practice schedule. Access to GP care is a major cause for concern that may be placing significant demand on Ambulance Services and Emergency Departments (ED).
  “I don’t believe we’re necessarily getting sicker as a nation but access to services is becoming a lot more bottlenecked…people will default to the easiest accessible service and the easiest service to sort of activate because they’re always going to come to you, is the Ambulance Service” (GP13).
iii. **Rural Vs Urban Practice**
  A common theme among respondents was workforce shortages and the implications of that. Respondents described the differences between rural and urban patient expectations and how this can affect their ability to see the sick people that need to be seen**.**
  “There are two different types of general practice in Ireland. There’s the urban-based and the rural based and I think the future viability of rural general practice is at a crossroads at the moment. I think the current model of smaller single/double-handed practices is in serious doubt” (GP08).

### Primary Care & Paramedicine Partnership

This theme explores the potential partnership between General Practice and Advanced Paramedics and potential roles for Advanced Paramedics within that collaboration. Out-of-hours services, Primary Care Team, and General Practice based Advanced Paramedic were discussed.

i. **Out-of-Hours Services**
  General Practitioners described the demands of providing out-of-hours services, house calls and nursing home visits and felt that this is a role where Advanced Paramedics would be beneficial. Respondents were very supportive of exploring this theme as a matter of urgency and felt that it should be prioritised for further exploration.
  “If anything, out of hours, should be acute care. So, the APs are probably, as a starting point, probably better suited to out of hours care, because it’s more acute patient care” (GP05)
ii. **Primary Care Team**
  General Practitioners felt that the Advanced Paramedics’ skillset could be seamlessly adapted and utilised in other areas such as chronic disease care, primary care team members or as part of a General Practice-Based Advanced Paramedic. Covid-19 has placed unprecedented demand on GP services resulting in more consultations being conducted over the phone.
iii. **General Practice Based Advanced Paramedic**
  An unanticipated theme emerged where General Practitioners felt Advanced Paramedics would be beneficial in providing telemedicine services and phone triage within the practice.
  “An awful lot of our time is taken up with telephone triage…Where could I see you guys coming in, literally taking the phone, rather than the receptionist…applying your clinical expertise to what you have been told. Having you make a decision on how you could help us. I could see that as something that we could explore” (GP03).

### From Theory to Tangibility

The third and final theme explores making the concept a reality and to examine and develop subthemes that emerged during this phase of the research study; subthemes included consensus, communication, innovation, employment and barriers and enablers.

“I think it’s very important that as General Practitioners, and Advanced Paramedics, that we take the lead on things and not wait for our hospital colleagues, that traditionally would be heading research, I think we need to start taking the lead and start putting our own data out there” (GP05).

i. **Consensus**
  Overall, General Practitioners were quite optimistic to the concept of introducing Advanced Paramedics into General Practice and portrayed a positive consensus to being directly involved in such a programme or pilot study. Respondents described their opinions around resistance or barriers that such a novel concept might face through their own organisation within the GP population and other government departments. Overall GPs did not believe that there would be much resistance from within their own profession and felt it would be mainly welcomed within General Practice.
  “I don’t think you’re going to be taking over a big patch from someone else, I suppose that would be one worry, the GPs would be saying, these guys are coming in on our patch, I don’t think so, I think we would have different roles, like I think the demand for healthcare work is justified? So, the work will be there anyway” (GP01).
ii. **Communication**
  Communication was discussed at length by most respondents. General Practitioners felt that like the issue of “*trust*” and “*being part of the team*” that communication was key. Respondents felt that they would be comfortable with telephone supervision until the AP and the GP had reached a mutual point of “*trust*”. This again was compared to the General Practitioners registrar example where the relationship would form over time between the GP and the AP. Respondents revisited this example and felt that it is a good basis for exploration. Respondents discussed what they believed to be the best approach to establish such a model and get it off the ground with most GPs volunteering to be part of the steering group or pilot study.
iii. **Innovation**
  On the topic of Advanced Paramedics working in out-of-hours services, respondents expressed an urgency and immediate desire to act on this initiative. Respondents that were aware of Paramedics working in General Practice in the United Kingdom (UK) and other jurisdictions thought this was something that should be explored in the Republic of Ireland. A common theme echoed by many respondents was the impact of Covid-19 on driving innovative change within the healthcare system.
  “I think you’re at a good time, you’re at a cusp in history where this coronavirus has got us all thinking differently. We’re doing things we never did before, never thought possible and there is the window, there is the moment in time where you can influence change and I think certainly we have heightened respect for the Paramedics” (GP08).
iv. **Employment**
  It is necessary to establish who the Advanced Paramedics would work for, either the HSE, Ambulance Service, out-of-hours services or GP practice. General Practitioners felt that this could be approached in a similar way to the practice Nurse where HSE funding is made available. Some respondents felt the AP should remain within the remit of the HSE/NAS but felt this model diluted the partnership and more importantly the “trust” between the GP and AP. They felt that this would lead to a disconnect. GPs unanimously cited the issue of “trust” and felt that this potential partnership would have to be treated like the General Practitioners registrar where the Advanced Paramedics would undergo a period of preceptorship and mentorship from the GP to support Advanced Paramedics transition into General Practice. Out-of-hours Co-op was also presented as an option for direct employment as was a private agency operating a General Practitioners’ contract agency in the mid-west. Various options were discussed but a common theme among all respondents was that these barriers could be overcome.
  “I certainly would have shared employment for an Advanced Paramedic. So, maybe several practices that would have employed an AP between them and share the cost. I would see a huge need for it in rural practices” (GP02).
v. **Barriers and Enablers**
  Key discussion points emerged during the data collection phase that respondents felt may be barriers and enablers to the process: remuneration, indemnity, and funding. Although these common themes were discussed at length, GPs believed that these issues could be overcome.

### Remuneration

General Practitioners are private contractors and self-employed and therefore viewed the Advanced Paramedic as another member of the team as one model. Another suggestion was to replicate the UK model where the AP is directly employed by the GP practice or between several practices within a region. The Community Paramedic pilot (CP) model was also discussed where the CP remains directly employed by the HSE/NAS and is dispatched through a central control room. *GP13* is familiar with this model and works directly with the Community Paramedic in the border regions. He felt that while the Community Paramedic model is working well, there was a disconnect with the Community Paramedic as they are not linked to the surgery. This was a common theme among responding GPs.

### Indemnity

Respondents felt that they would have to have a connection with the Advanced Paramedic if they were treating patients on their behalf. The issue of “*trust*” resurfaced and “*being part of the team”* to be important to the success of any potential model. Indemnity was less of an issue in relation to out-of-hours where respondents felt this was a concept that could be explored immediately as the Co-op was a private contractor and did not envisage indemnity an issue once the AP would be working as part of a GP led team.

“I don’t see indemnity as a problem as we are private contractors with our own insurance and the AP could be covered under that” (GP01).

#### Funding

Respondents had various approaches to this concept. It was presented that a directly funded program by the GP practice may deviate from the original concept as private practice is based on remuneration. They felt the Advanced Paramedic could be utilised in areas that would make money for the practice and that this would be counterproductive and against the ethos of the original design. Another approach was HSE funding like the practice nurse. The current pilot Community Paramedic program was also discussed as an example where the AP would remain within the remit of the HSE/NAS utilising their vehicle, equipment, and communications devices; this replicated issues of disconnect, trust and not being part of the team. Respondents echoed their view, that a model should replicate the GP registrar training so far as the Advanced Paramedic would be part of the practice team, attached to that practice under the guidance of the GP. None of the respondents favoured an ad-hoc approach, like the current Community Paramedic pilot model where access to the Community Paramedic is disjointed or diluted and shared with other practices outside the area.

## Discussion

### Summary of findings

The primary aim of this study was to explore General Practitioners’ attitudes and opinions for the potential of integrating Advanced Paramedics into Primary Care and General Practice. This research suggests that GPs are willing to explore this option and to recognise the potential opportunities for collaboration. GPs were overwhelmingly positive towards the concept of integrating Advanced Paramedics into General Practice and recognised the potential for such a program, providing numerous examples where they could see areas for collaboration. The most common roles or tasks were in home visits, nursing homes and particularly out-of-hours services. Most respondents envisaged a clinical role within the surgery including patient assessment, management, and triage. GPs do not believe there would be much resistance from within General Practice as they recognise their workload has far exceeded their capacity. International evidence describes the evolution of Paramedic practice far beyond the traditional norm. Paramedics in the United Kingdom, Australia, and Canada are practicing within a variety of healthcare settings including home visits, nursing homes, minor injury units, urgent care units, mental health and forensic health facilities, acute hospital trusts, emergency departments, primary care, and general practice [31]. While there is limited quality data available on the safety and efficacy of Paramedics working within these environments, patient satisfaction does appear to be positive as access to GP services becomes increasingly more difficult [32, 33]. There have been numerous reports documented within the literature describing the challenges facing General Practice including increased workload and burnout. These testimonies have provided an extraordinary insight into their personal lives [1]. The General Practitioners’ views and opinions are expert within their field. Their accounts are detailed and information-rich with great meaning. This is a report of the views of a cross-section of the study population [11]. Interviews were widely advertised through e-mail to a national General Practitioners network (Irish Rural, Island and Dispensing Doctors). The respondents are broadly representative of the study population across the island of Ireland. In this study, there was some disparity between GP’ survey answers about Advanced Paramedic skills transferability and the answers given in the interview setting. A less extensive set of conditions was mentioned in the interviews. This may be because many GPs were focused on barriers to providing services and/or unfamiliar with the skills of Advanced Paramedics described in the literature. Interestingly, there was an unanticipated emphasis on the role of the AP in providing telemedicine services and phone triage within the practice. This could be due to the timing of the interviews during the COVID-19 pandemic and its consequential increased use of telemedicine. The findings within the quantitative data required further investigation through qualitative interviews, where GPs replicated their unanimous support for collaboration with Advanced Paramedics that was demonstrated utilising two separate data sets, quantitative and qualitative methodologies. The mixed methodology approach facilitated a more robust approach into this finding that may not have been possible using a mono-methodology approach [26, 34].

### Future research and cost implications

This research has established the views of General Practitioners and their support for collaboration and change. Community Paramedic programmes are beneficial although will take significant time and resources to appropriately implement. International evidence has demonstrated treating patients within their own homes and community increases patient satisfaction and reduces demand on healthcare capacity and expenditure [21]. This contributes to a reduction in overall healthcare expenditure, GP workload, ambulance transport, emergency department attendance and hospital admission. Advanced Paramedics are ingrained in the community and can play a larger role in healthcare delivery, especially in rural Ireland. Future research would be required to examine the views of the Advanced Paramedics and other stakeholders to gain an insight into their position(s). A working group or *delphi* process consisting of key stakeholders from within the disciplines of general practice, ambulance service, health service, department of health, hospital groups, patient advocate groups, and representative unions is required. This could examine the feasibility of such a project and hopefully move it to pilot study phase. A pilot study where an Advanced Paramedic joins a GP team for several months to participate in the suggested activities identified in this study would also be a worthwhile program of research that would provide evidence of whether Advanced Paramedics are effective in General Practice. It is envisaged the cost of implementing this concept would be minimal as this study suggests utilising existing resources more efficiently. Advanced Paramedics are already established within the healthcare system with primary focus on emergency medicine [35]. Efficient use of AP resources could reduce overall expenditure, ambulance transport, ED attendance and hospital admission [20]. The funding of Advanced Paramedic posts in General Practice in Ireland is another factor that requires consideration. Reimbursement for Advanced Paramedics in General Practice does not currently exist in Ireland. Likely avenues for funding could include the Health Service Executive. The generalisability of the study findings to other countries will vary as different health systems have different funding models for General Practice and differences in the licensing and regulating of Paramedicine roles.

### Recommendations for practice

Based upon General Practitioners testimony the key recommendations for practice from this study are; Advanced Paramedics may be underutilised and may have a greater role to play within the wider healthcare system like their contemporaries within the United Kingdom. Community Paramedic programmes will take several years to implement and reach a scale of service that delivers results at a population level. Advanced Paramedics are already practicing within the community and with minimal adjustments as outlined below could take on the proposed new roles within General Practice. General Practitioners have provided testimony where they see APs’ current skill set applied but aimed towards General Practice rather than emergency practice under the guidance of the GP. Urinary retention catherization, fluid therapy and phlebotomy were some of the common clinical scenarios where the GP believed APs could provide much needed supports that could be utilised in out-of-hours services, home visits and nursing homes. Although catheterisation would be a new skill for Advanced Paramedics, it requires minimal training. The paramedic registration body in Ireland, Pre-hospital Emergency Council (PHECC) have developed draft “treat and discharge” protocols that could be implemented safely without delay under the auspices of the local GPs [35]. General Practitioners have described how they envisage APs attending house calls on their behalf providing assessments and care while communicating with the GP. This approach has been shown to be beneficial for patients while reducing ambulance conveyance, Emergency Department attendance, hospital admission and healthcare costs. While not the focus of this study, support for rural General Practice in Ireland requires immediate action. However, this will be achieved, the implications of inaction in this area will restrict future access to services that will disproportionately impact elderly patients with chronic disease and co-morbidities that will have serious ramifications on future healthcare planning and expenditure.

### Strengths and limitations

Whilst data saturation was achieved within this study relative to the research question, it is only representative of one key stakeholder. Due to study constraints, the voice of Advanced Paramedics has not been represented within this research. It would be important to establish the views and opinions of Advanced Paramedics and that will be the focus of future studies. General Practitioners were overwhelmingly supportive towards the concept of integrating Advanced Paramedics into General Practice: no dissenting voice was discovered among this cohort. This finding was replicated over two separate data collection methodologies. This may be due to selection bias amongst GPs that have an interest in the topic and are more likely to volunteer to take part in the qualitative interviews. This may be improved using other methodology in the future such as seeking to get a consensus approach to an implementation strategy with a larger, more diverse sample of GPs. This may be an area that requires further research.

## Conclusion

The findings of this study have highlighted the extent of the increasing, intolerable burden facing rural General Practitioners in Ireland, and have provided extensive testimony of their wholehearted support for the idea of further exploring the usefulness and feasibility of integrating Advanced Paramedics into various areas of their practice. Challenges will have to be overcome with much more work. What is evident is that General Practitioners in rural Ireland are prepared to engage in that process and overcome the barriers of remuneration, indemnity, and funding. Further stakeholder engagement, including understanding the views of Advanced Paramedics and a pilot feasibility initiative are the suggested next steps. The Covid-19 pandemic has demonstrated what can be achieved when a crisis necessitates an innovative response. Rural General Practice in Ireland is facing a potential crisis of grave magnitude that will require a similar innovative response. Perhaps the time is right to act upon this momentum and seize this opportunity before it is too late.

## Supplementary Information

**Additional file 1.**

**Additional file 2.**

**Additional file 3.**

**Additional file 4.**

**Additional file 5.**

## Data Availability

All data and material used and analysed during the current study will be available from the corresponding author on request.

## Abbreviations

AP: Advanced Paramedic
CP: Community Paramedic
ED: Emergency Department
EU: European Union
GP: General Practitioner
HSE: Health Service Executive
NAS: The National Ambulance Service
NCHD: Non-Consultant Hospital Doctor
PCRS: Primary Care Reimbursement Service
PHECC: Pre-Hospital Emergency Care Council
ROI: Republic of Ireland
UK: United Kingdom
