# Supplementary material for: Introducing Advanced Paramedics into the rural general practice team in Ireland – general practitioners attitudes
Source: BMC Prim Care. 2022 May 26;23:130. doi: 10.1186/s12875-022-01740-9 (PMC9134982; doi:10.1186/s12875-022-01740-9)
Supplement: Supplementary file 1 — Additional file 1. [file 12875_2022_1740_MOESM1_ESM.pdf]

General Practitioner Questionnaire

(Please circle as appropriate)

Thank you for taking the time to answer this questionnaire. This research aims to examine potential mechanisms that can support General Practice (GP) by shifting some appropriate tasks towards alternative existing healthcare providers Advanced Paramedics (APs), within primary care that may be of mutual benefit to both GPs, APs and their patients.

1.	Age	25-30	30-35	35-40	40-45	45-50	50-55	55-60	60-65	>65

2.	Gender	Female	Male

3.	Geographical make-up of your practice	Urban	Rural	Mixed

4. Are you involved in GP training scheme within your practice?	Yes	No

5. Approximately, how many patients are currently registered with you?	

6. Are you familiar with Advanced Paramedics scope of clinical practice?	Very unfamiliar	Unfamiliar	Undecided	Familiar	Very Familiar
	1	2	3	4	5

7. To support GP's in practice would you consider exploring the concept of shifting some of the following tasks to Advanced Paramedics within their current scope of clinical practice? (Please circle as appropriate)	Strongly Disagree 1	Disagree 2	Undecided 3	Agree 4	Strongly Agree 5
1. Perform a detailed cardiovascular examination including Hx taking, recognition of common cardiac conditions including risk factors & common causes of chest pain & ACS	1	2	3	4	5
2. Acute Myocardial Infraction/ACS management including intravenous cannulation & appropriate drug therapy i.e analgesic, nitrates, anticoagulation, thrombolytic therapy ect.	1	2	3	4	5
3. Perform a detailed respiratory examination, including Hx taking, recognition of common respiratory conditions, asthma, COPD, pulmonary oedema ect.	1	2	3	4	5
4. Manage acute respiratory emergencies including severe asthma, exacerbation of COPD, Acute pulmonary oedema	1	2	3	4	5
5. Perform a detailed gastro-intestinal examination, including Hx taking, differential diagnosis of abdo pain, recognition of common GI conditions based on Hx & examination.	1	2	3	4	5
6. Manage acute abdominal emergencies including administration of appropriate anti-emetics and analgesics.	1	2	3	4	5
7. Perform a detailed neurologic examination, including Hx taking recognition of common conditions including migraine, CVA, intracranial haemorrhage ect & appropriately manage acute emergency presentations.	1	2	3	4	5
8. Perform patient assessments & appropriate treatment in the surgery	1	2	3	4	5
9. Perform patient assessment & appropriate treatment at patients home if requested	1	2	3	4	5
10. Recognition/verification of death	1	2	3	4	5

8. In your opinion what additional knowledge/competencies would an Advanced Paramedic require to be most beneficial to support you in your practice? e.g catheterisation, phlebotomy, diagnostic tests, etc.)<br><br>(Please print answer)
1.
2.
3.
4.
5.
6.

Thank you,

Fintan Feerick, EMT-AP, MSc

Paramedic Studies, Graduate Entry Medical School,

University of Limerick

fintan.feerick@ul.ie

Mob: 087 6659334
