# Supplementary material for: Introducing Advanced Paramedics into the rural general practice team in Ireland – general practitioners attitudes
Source: BMC Prim Care. 2022 May 26;23:130. doi: 10.1186/s12875-022-01740-9 (PMC9134982; doi:10.1186/s12875-022-01740-9)
Supplement: Supplementary file 2 — Additional file 2. [file 12875_2022_1740_MOESM2_ESM.pdf]

Semi Structured Interview Guide

1. How do you see the future sustainability of rural general practice in Ireland?
2. What are the biggest challenges that you face in your rural practice? Develop these issues.
3. What type of supports do you need to sustain future services?
4. Do you think APs could provide some supports, if so, how?
5. How do you think such a model could be implemented & funded?
6. Would you consider directly employing an AP within your practice or as a co-op among other practices in your region?
7. Do you provide home visits or out-of-hours services? Could APs take on some of these tasks?
8. If so, how could you see this working, i.e unsupervised, phone supervision, bodycams, telemedicine ect.
9. What is your opinion on APs prescribing? How might this work, restricted practice, antibiotics ect.
10. What do you think might be the biggest barriers to implementing such a model in rural general practice in Ireland? Develop issues.
11. Would you be willing to provide training or mentoring to an Advanced Paramedic to develop their knowledge and skills?
12. Is there anything else that you would like to add?

Ask for personal cases and examples where possible
