# Supplementary material for: Introducing Advanced Paramedics into the rural general practice team in Ireland – general practitioners attitudes
Source: BMC Prim Care. 2022 May 26;23:130. doi: 10.1186/s12875-022-01740-9 (PMC9134982; doi:10.1186/s12875-022-01740-9)
Supplement: Supplementary file 3 — Additional file 3. [file 12875_2022_1740_MOESM3_ESM.pdf]

Participant Information Sheet and Consent form

Advanced Paramedics in General Practice-An Exploratory Study?

Summary

Healthcare policy in Ireland is in a state of transition, to reduce pressures on the acute hospital setting, more patients and services are being diverted back into general practice. Current and future predictions suggest that there are insufficient number of existing GPs available to meet this increased demand, especially in rural areas. Advanced Paramedics are an untapped resource that may have the capability to provide essential support to rural general practice that could be mutually beneficial for both parties and their patients alike.

Introduction to the study

The aim of this study will be to explore if GPs would consider the concept of shifting certain tasks towards Advanced Paramedics to help sustain the future of rural general practice in Ireland.

What will I be asked to do?

If you agree/consent to be involved in this study, you will be invited to participate in a short interview (max 40 mins) to gain an insight into your attitude and opinion towards exploring the concept of integrating Advanced Paramedics into general practice and the potential associated ramifications.

What are the benefits?

The potential benefits of taking part in this study may identify that Advanced Paramedics may be in a position to provide essential supports to help sustain the future of rural general practice in Ireland, that may be mutually beneficial to both parties and their patients alike.

What are the risks?

There is no risk associated to you if you agree to participate in this study. It is anticipated to take no more than 40 minutes to complete the interview which will be conducted at a time and place of your choosing. Due to current Covid-19 restrictions this can take place via telephone or alternative internet-based media (Zoom, Microsoft meeting, WhatsApp etc). You may withdraw from the study at any point and any data collected at this point will be deleted.

Anonymity
All information gathered will be treated in the strictest of confidence and will be collected anonymously. To ensure this, your name will be removed from all the data and replaced with an ID number. Only the named investigator (Fintan Feerick) on the project will have access to the ID number files and these will be in no way linked to your identity.

Data Protection
It is a legal requirement under data protection law that I supply you with the following information in Appendix 1. If there is anything you do not understand you can ask me to explain.

Ethics Approval.
This research study has received Ethics approval from the Education and Health Sciences Research Ethics Committee, REC Ref: 147/18.

If you have any concerns about this study and wish to contact someone independent, you may contact: Mr. Pat Dillon, Chair Education and Health Sciences Research Ethics Committee, EHS Faculty Office, University of Limerick. Tel (061) 482519

Thank you for reading.
If you have any further questions about the study or if you want to opt out of the study, you may do so at any time of your choosing. If you need any further information now or at any time in the future, please contact: fintan.feerick@ul.ie

Yours sincerely,

Fintan Feerick

Paramedic Studies
Scoil an Leighis **| School of Medicine**
Dámh an Oideachais & na nEolaíochtaí Sláinte **| Faculty of Education & Health Sciences**
Ollscoil Luimnigh **| University of Limerick**
Luimneach, V94 T9PX **| Limerick, V94 T9PX**
Éire**|Ireland**

PARTICIPATION CONSENT FORM

**Study title: Advanced Paramedics in General Practice-**<br>**An Exploratory Study?**

I have read and understood the **Information Leaflet** about this research project. The information has been fully explained to me and I have been able to ask questions, all of which have been answered to my satisfaction.	**Yes** ☐	**No** ☐
I understand that I don't have to take part in this study and that I can opt out at any time. I understand that I don't have to give a reason for opting out and I understand that opting out won't affect my future medical care.	**Yes** ☐	**No** ☐
I consent to take part in this research study having been fully informed of the risks, benefits and alternatives.	**Yes** ☐	**No** ☐
I am aware that my participation in this study will be **audio recorded** and I agree to this. However, if I feel uncomfortable at any time, I can ask that the recording equipment be switched off. I understand that I can ask for a copy of my recording. I understand what will happen to the recordings once the study is finished.	**Yes** ☐	**No** ☐
I give informed explicit consent to have my data processed as part of this research study.	**Yes** ☐	**No** ☐
I consent to be contacted by researchers as part of this research study	**Yes** ☐	**No** ☐

| | |

Participators Name (Block Capitals) | Participators Signature

| Date

To be completed by the Principal Investigator or nominee. I, the undersigned, have taken the time to fully explain to the above participant the nature and purpose of this study in a way that they could understand. I have explained the risks involved as well as the possible benefits. I have invited them to ask questions on any aspect of the study that concerned them.

| |

Name (Block Capitals) | Signature | Date

Appendix 1

Data Protection

It is a legal requirement under data protection law that I supply you with the following information. If there is anything you do not understand you can ask me to explain.

1. I will be using the anonymous information from your participation in this study.
2. The legal basis under which I will be processing your data is for legitimate interests and for scientific research purposes.
3. The principal investigator, Mr. Fintan Feerick, will have access to your anonymous data along with basic information (age and gender). You will not be identifiable in any of these results. This information will be held with the appropriate safeguards by the principle investigator.
4. The data will be stored securely for a period of 5 years and then destroyed. It is required for research validity to store the data for this period of time.
5. I do not anticipate any risk to you as a result of the data processing as I am only interested in anonymous information.
6. You have a right to request access to your data and a copy of it.
7. You have the right to withdraw consent to your personal data being used in this research project and to have your personal data deleted. You have a right to restrict or object to processing. You will be able to do this by contacting Mr. Fintan Feerick (see contact details below.
8. You have a right to lodge a complaint with the Data Protection Commissioner.
9. You have a right to have any inaccurate information about you corrected or deleted.
10. You have a right to data portability, meaning you have a right to move your data from one controller to another in a readable format.
11. Your anonymous data will be kept locked in a filing cabinet in the office of the principal investigator for the required 5-year period, then destroyed. Only anonymous data will be shared with the larger supervisory research team and in my reporting.
