# Supplementary material for: Introducing Advanced Paramedics into the rural general practice team in Ireland – general practitioners attitudes
Source: BMC Prim Care. 2022 May 26;23:130. doi: 10.1186/s12875-022-01740-9 (PMC9134982; doi:10.1186/s12875-022-01740-9)
Supplement: Supplementary file 4 — Additional file 4. [file 12875_2022_1740_MOESM4_ESM.pdf]

Feidhmeannacht na Seirbhíse Sláinte
Health Service Executive

26th June 2019

Re: Research Approval

Dear Mr Feerick,

Following on from your recent application to conduct research, the National Ambulance Service Research Committee hereby gives approval for you to conduct research on the following topic ”Task Shift Analysis in Primary Care Workforce Planning in Ireland”.

We would be most grateful if you could submit a final copy of your research to NAS for our library.

Yours sincerely,

____

Emily Mahon
NAS Business Manager
On behalf of the NAS Research Committee
