# Supplementary material for: Introducing Advanced Paramedics into the rural general practice team in Ireland – general practitioners attitudes
Source: BMC Prim Care. 2022 May 26;23:130. doi: 10.1186/s12875-022-01740-9 (PMC9134982; doi:10.1186/s12875-022-01740-9)
Supplement: Supplementary file 5 — Additional file 5. [file 12875_2022_1740_MOESM5_ESM.pdf]

Ospidéil OL
UL Hospitals

Ospidéal na hOllscoile, Luimneach
University Hospital Limerick,

Quality & Safety Department,
UL Hospitals Group,
HSE, Unit 2,
Loughmore Avenue,
Raheen Business Park,
Limerick, V94 P7X9
Tel: 061 482519
joanne.oconnor@hse.ie

11th December, 2018.

Mr Fintan Feerick,
Paramedic Studies,
Room ICG 022,
Graduate Entry Medical School
University of Limerick
Limerick.

Re: Protocol Title:
Task Shift Analysis in Primary Care Workforce Planning in Ireland.
REC Ref: 147/18

Dear Mr Feerick,

I am in receipt of your proposal as above submitted for review by our Research Ethics Committee. I have reviewed the contents of same.

I wish to advise that I have given this study Chairperson ethical approval. You should note that your study cannot commence until you also receive AON approval which will issue from the Quality and Safety Department shortly. You are obliged to inform us as soon as your study is completed or if it terminates early for any reason.

I should take this opportunity to remind you of the importance of compliance with Data Protection Legislation and guidance at all times.

While this letter is granting ethical approval for your study, it is also necessary that you ensure you have local site access approval to conduct this research.

I wish you every success with your study.

Yours sincerely,

Pat Dillon,
Consultant Anaesthetist,
Chairperson, Research Ethics Committee.

ULH
Caring, Courteous and Professional

Feidhmeannacht na Seirbhíse Sláinte
Health Service Executive
